# Arginine metabolic endotypes related to asthma severity

**DOI:** 10.1371/journal.pone.0183066

**Published:** 2017-08-10

**Authors:** Weiling Xu, Suzy A. A. Comhair, Allison J. Janocha, Abigail Lara, Lori A. Mavrakis, Carole D. Bennett, Satish C. Kalhan, Serpil C. Erzurum

**Affiliations:** 1 Department of Pathobiology, Lerner Research Institute, Cleveland Clinic, Cleveland, Ohio, United States of America; 2 Respiratory Institute, Cleveland Clinic, Cleveland, Ohio, United States of America; National and Kapodistrian University of Athens, GREECE

## Abstract

**Aims:**

Arginine metabolism *via* inducible nitric oxide synthase (iNOS) and arginase 2 (ARG2) is higher in asthmatics than in healthy individuals. We hypothesized that a sub-phenotype of asthma might be defined by the magnitude of arginine metabolism categorized on the basis of high and low fraction of exhaled nitric oxide (F_E_NO).

**Methods:**

To test this hypothesis, asthmatics (*n* = 52) were compared to healthy controls (*n* = 51) for levels of F_E_NO, serum arginase activity, and airway epithelial expression of iNOS and ARG2 proteins, in relation to clinical parameters of asthma inflammation and airway reactivity. In parallel, bronchial epithelial cells were evaluated for metabolic effects of iNOS and ARG2 expression *in vitro*.

**Results:**

Asthmatics with high F_E_NO (≥ 35 ppb; 44% of asthmatics) had higher expression of iNOS (*P* = 0.04) and ARG2 (*P* = 0.05) in the airway, indicating F_E_NO is a marker of the high arginine metabolic endotype. High F_E_NO asthmatics had the lowest FEV_1_% (*P* < 0.001), FEV_1_/FVC (*P* = 0.0002) and PC_20_ (*P* < 0.001) as compared to low F_E_NO asthmatics or healthy controls. Low F_E_NO asthmatics had near normal iNOS and ARG2 expression (both *P* > 0.05), and significantly higher PC_20_ (*P* < 0.001) as compared to high F_E_NO asthmatics. *In vitro* studies to evaluate metabolic effects showed that iNOS overexpression and iNOS+ARG2 co-expression in a human bronchial epithelial cell line led to greater reliance on glycolysis with higher rate of pyruvate going to lactate.

**Conclusions:**

The high F_E_NO phenotype represents a large portion of the asthma population, and is typified by greater arginine metabolism and more severe and reactive asthma.

## Introduction

Asthma is a chronic inflammation of the airways that is characterized by airway reactivity. Nitric oxide (NO), generated by inducible NO Synthase (iNOS; EC 1.14.13.39) expressed in the airway epithelium, where it catalyzes the conversion of arginine to NO and citrulline, is typically higher in asthmatics than in healthy populations [1–4]. We [1, 5, 6] and others [7–13] found that levels of arginine and arginase 2 (ARG2; EC 3.5.3.1), which catabolizes arginine to ornithine and urea, are also higher in the airway of asthmatics as compared to healthy controls. Recently we reported that sustained NO production in asthma is dependent upon the cell autonomous citrulline-arginine-NO cycle [6]. ARG2, by delivering ornithine into the mitochondria, provides nitrogen for the citrulline-arginine-NO cycle in order to sustain the high NO production while also supporting cellular bioenergetics and the inflammatory state [6].

In this context, although NO is higher in asthmatic populations, there is a wide variability in fraction of exhaled NO (F_E_NO) in individuals with asthma; some have F_E_NO levels in a normal range. Little is known of the high and low F_E_NO endotypes, and the bioenergetic effects of high arginine metabolism on asthma. Guidelines for asthma care define a cut-point of 35 ppb F_E_NO for asthma control and assessment of inflammation [4, 14]. Here, we hypothesized that a sub-phenotype of asthma could be defined by the magnitude of arginine metabolism identified on the basis of high and low F_E_NO levels.

## Materials and methods

### Study population

The clinical characteristics of the study population are listed in Table 1. Some of the study subjects had participated in studies reported previously [5, 6]. Asthma was verified based upon American Thoracic Society guidelines, which include positive methacholine challenge test and/or reversible airflow obstruction. The severity of asthma was classified as mild intermittent/persistent (mild), moderate persistent (moderate) and severe persistent (severe) based on National Asthma Education and Prevention Program (NAEPP) guidelines [15]. Healthy controls lacked cardiopulmonary symptoms and had normal spirometry and negative methacholine challenge. Exclusion criteria for both asthmatics and healthy controls included age less than 18 years, pregnancy, current smoking, smoking within the past year, or former smokers with ≥ 5 pack-year total history. Spirometry was performed with an automated spirometer, and F_E_NO was measured by an online method at a constant flow rate of 50 ml/second according to the standards published by the American Thoracic Society. A subgroup of participants underwent bronchoscopy for endobronchial brushing and for bronchoalveolar lavage (BAL). All studies were approved by the Cleveland Clinic Institutional Review Board (IRB # PPG8351). All subjects were recruited from Cleveland Clinic and gave written informed consent by signing a consent document approved by the Cleveland Clinic Institutional Review Board.

**Table 1:** Clinical features of study participants.

Characteristics	Healthy Controls	All Asthmatics	P*	Asthma Severity			
				Mild	Moderate	Severe	P**
N	51	52		27	18	3	
Mean age, yr	37 ± 1	38 ± 1	0.8	37 ± 2	41 ± 1	36 ± 7	0.4
Gender, M/F	18/33	23/29	0.3	10/17	10/8	1/2	0.4
BMI, kg/m^2^	27.8 ± 0.9	29.0 ± 0.8	0.17	27.4 ± 1.0	30.6 ± 1.1	34.5 ± 4.0	0.03
Ethnicity, C/AA/other	24/23/4	25/20/7	0.4	15/8/4	7/9/2	1/2/0	0.7
Lung function							
FEV_1_% predicted	96 ± 1	82 ± 2	<0.001	94 ± 1	71 ± 1	52 ± 4	<0.001
FEV_1_/FVC	0.80 ± 0.01	0.74 ± 0.01	0.0002	0.77 ± 0.01	0.71 ± 0.02	0.54 ± 0.03	<0.001
PC_20_, mg/ml	NR^#^	4.6 ± 1.3	<0.001	6.3 ± 1.9	2.3 ± 1.5	0.26 ± 0	0.2
IgE, IU/ml	76 ± 21	291 ± 51	0.0004	278 ± 74	310 ± 86	290 ± 29	0.9
Medication (*n*)							
Corticosteroids (no/yes)	51/0	22/30		15/12	3/15	0/3	0.006
Inhaled (no/yes)	51/0	22/30		15/12	3/15	0/3	0.006
Oral (no/yes)	51/0	52/0		27/0	18/0	3/0	
Injected (no/yes)	51/0	52/0		27/0	18/0	3/0	
Xolair (no/yes)	51/0	52/0		27/0	18/0	3/0	
Leukotriene receptor antagonists (no/yes)	51/0	41/11		22/5	14/4	2/1	0.8

Mean ± SEM;

Definition of abbreviations: Mild, mild intermittent/persistent; Moderate, moderate persistent; Severe, severe persistent; M, male; F, female; BMI, body mass index; C, Caucasian; AA, African American; FEV_1_, Forced expiratory volume in 1 second; FVC, Forced vital capacity; PC_20_, provocative concentration of methacholine causing a 20% fall in FEV_1_;

*P value, asthma *vs*. controls;

**P value, ANOVA of mild, moderate, severe asthma;

^#^non-reactive.

### Arginase activity

Arginase activity was determined, using 50 μl aliquots of serum, by the conversion of [^14^C]guanidino-L-arginine to [^14^C]urea, which was converted to ^14^CO_2_ by urease and trapped as Na_2_^14^CO_3_ [5, 16].

### Western analyses

Whole cell lysates were prepared as previously described [17]. Total protein was measured using the Pierce Coomassie Plus Protein Assay (Thermo Fisher Scientific, Waltham, MA). For human bronchial epithelial cells, the volumes of whole cell lysate loaded per lane were adjusted to have similar Cytokeratin expression after 5 μl lysates were pre-run to measure Cytokeratin, because Cytokeratin confirms epithelial cells obtained by airway brushing. Equal amounts of protein (80 μg/lane) were loaded per lane for samples from BET1A cells, while Enolase was used as a loading control. Proteins were separated by electrophoresis on a 4–15% Tris-HCl precast gel (Bio-Rad Lab, Hercules, CA) and transferred onto polyvinylidene difluoride membranes (PVDF, Millipore Corporation, Bedford, MA). Rabbit anti-iNOS (sc-651, 1:400), ARG2 (sc-20151, 1:600) and Enolase (sc-15343, 1:1000) polyclonal Ab (Santa Cruz Biotechnology, Santa Cruz, CA), and mouse anti-Cytokeratin (M3515, 1:400) monoclonal Ab (DAKO North America, Carpinteria, California) were used in western analyses.

### Cell culture

BET1A cells, a human bronchial cell line transformed by SV40 T antigen, were cultured in serum-free LHC-9 medium (Thermo Fisher Scientific) on pre-coated plates.

### Transient transfection

Using Lipofectamine 2000 Reagent (Invitrogen Corp, Carlsbad, CA), BET1A cells were transiently transfected for 6 hours with human iNOS expression plasmid (iNOS, CCF37) [18, 19], ARG2 expression plasmid (ARG2, CCF88) [6], or an empty control vector, or co-transfected with iNOS plasmid and ARG2 plasmid (iNOS+ARG2). After transfection, cells were treated with 3 mM NO synthase inhibitor N-nitroarginine methyl ester (L-NAME) or left untreated. 48 hours after transfection, the cells were harvested for further analysis.

### ATP and lactate content

ATP in cell lysates was measured by using a luciferase-based luminescence assay kit (PerkinElmer, Waltham, MA). Lactate concentration in BAL was determined with Lactate Assay Kit (BioVision, Milpitas, CA).

### Measurement of oxygen consumption rate (OCR) in cells

OCR was measured using the Seahorse Extracellular Flux (XF24) Analyzer (Agilent Technologies, Santa Clara, CA). Seahorse assay media (DMEM without glucose, L-glutamine, phenol red, sodium pyruvate, and sodium bicarbonate [Sigma-Aldrich, St. Louis, MO] prepared with 6 mM glucose, 1.85 g/l sodium chloride, 1 mM sodium pyruvate, and 15 mg/l phenol red) was freshly supplemented with 2 mM L-glutamine and the pH adjusted to 7.35 with sodium hydroxide. BET1A cells were detached, washed 3 times with Seahorse assay medium, and resuspended in assay medium. 6×10^5^ cells in 150 μl assay medium were added to the wells of a Seahorse cell plate and adhered using BD Cell-Tak (BD Biosciences, Bedford, MA) according to Seahorse protocol for nonadherent cells, with 2 or 4 wells per plate left empty for background correction. The plate was incubated in a 37°C non-CO_2_ incubator for 25 minutes. Additional assay medium was added to bring the final per-well volume to 500 μl and the plate incubated in a 37°C non-CO_2_ incubator for an additional 15 minutes. The plate was then transferred to the Seahorse XF24 Analyzer for analysis. Once in the XF24, BET1A cells underwent a MitoStress test (basal measurement of oxygen consumption followed by successive treatments with oligomycin A [0.5 μM], FCCP [carbonyl cyanide-ρ-trifluoromethoxyphenylhydrazone; 0.5 μM], and rotenone and antimycin A [1.5 μM]). All MitoStress OCR measures were done 3 times in a 3-2-3-minute mix-wait-measure cycle.

### Radioisotope studies of glucose metabolism in cells

The rate of oxidation of glucose to CO_2_ and the rate of glycolysis (glucose to lactate) were measured by incubating 1−2×10^6^ BET1A cells in LHC9 medium containing 6 mM glucose with [^14^C]-glucose in an atmosphere of 95% O_2_ and 5% CO_2_ in an airtight Erlenmeyer flask [6]. Cells were incubated for 3 hours. The CO_2_ generated was flushed by adding sodium bicarbonate and sulfuric acid into the medium. CO_2_ trapped in hyamine was counted in a scintillation counter. Lactate was separated by ion exchange chromatography and radioactivity in the isolated organic acids, mostly lactate, was measured using a scintillation counter. The rate of conversion of glucose to lactate was calculated using precursor-product relationship.

### Statistics

Data are shown as mean ± SEM. All statistical comparisons were performed using the Student’s t-test, Wilcoxon nonparametric analyses or ANOVA as appropriate. Relationships between groups were analyzed using Multivariate Pairwise Correlation. The level of significance for *P* was chosen at 0.05. All data were analyzed with statistical program JMP Pro 10 (SAS Institute, Cary, NC).

## Results

### Clinical population

The study population included 51 healthy controls and 52 individuals with asthma. The clinical characteristics of the study subjects are displayed in Table 1. Asthmatics had airway reactivity to methacholine and mild to moderate airflow limitation as measured by forced expiratory volume in 1 second (FEV_1_) and the ratio of FEV_1_ to forced vital capacity (FVC)(FEV_1_% predicted, Control 96 ± 1, Asthma 82 ± 2, *P* < 0.001; FEV_1_/FVC, Control 0.80 ± 0.01, Asthma 0.74 ± 0.01, *P* = 0.0002). Asthmatics were divided into mild, moderate and severe asthma according to FEV_1_ (FEV_1_% predicted, mild, ≥ 80, *n* = 27, moderate, > 60 but < 80, *n* = 18, severe, ≤ 60, *n* = 3)[15]. Individuals with severe asthma had the highest body mass index (BMI)(ANOVA *P* = 0.03) and worst lung function (*P* < 0.001) as compared with mild and moderate asthmatics (Table 1). Asthma was stable and controlled by inhaled corticosteroids without recent exacerbations and without recent systemic corticosteroids use (Table 1). Not all individuals underwent all experimental studies; the numbers of subjects assessed are provided with each experiment and result. Medications were withheld for one day prior to testing.

### Arginine metabolic endotypes related to clinical asthma phenotypes

Previously, we [1–3, 5, 6] and others [7–12] reported that asthmatics had higher levels of serum arginase activity and had higher F_E_NO. Using untargeted analysis, we observed a nitric oxide-related metabolomic endotype in asthma [3]. We also reported that the high rate of NO production was dependent upon induction of ARG2 and upon cell autonomous arginine-NO-citrulline cycle [6]. To examine whether arginine metabolic endotypes were informative of clinical asthma phenotypes, F_E_NO levels, serum arginase activity, and the expression of iNOS and ARG2 in freshly obtained airway epithelium were evaluated in asthmatics and healthy controls. As reported previously [1–5], asthmatics had significantly higher F_E_NO and greater serum arginase activity when compared with healthy controls [F_E_NO ppb, Control 19 ± 1, *n* = 51, Asthma 43 ± 5, *n* = 52, *P* < 0.001; Arginase activity μmol/ml/h, Control 0.30 ± 0.07, *n* = 3, Asthma 0.52 ± 0.08, *n* = 14, *P* = 0.05](S1 Table). Both iNOS and ARG2 proteins were generally expressed at higher levels in the airway epithelium of asthmatics compared with healthy controls [iNOS protein expression relative to cytokeratin expression, control 1.1 ± 0.2, *n* = 5, asthma 23.9 ± 13.8, *n* = 10, *P* = 0.01; ARG2 protein expression relative to cytokeratin expression, control 1.0 ± 0.1, *n* = 5, asthma 3 ± 1.3, *n* = 9, *P* = 0.03](S1 Table). Severe asthmatics had the highest F_E_NO levels (ANOVA *P* = 0.003), but similar expression of iNOS and ARG2 proteins in the airway epithelium (both ANOVA *P* > 0.05) as compared to individuals with mild and moderate asthma (S1 Table). Similar to previously reported suppressive effects of corticosteroids on iNOS/NO [1], asthmatics using inhaled corticosteroids tended to have lower F_E_NO levels than asthmatics not on corticosteroids (*P* = 0.17)(S2 Table). However, serum arginase activity and airway expression of iNOS and ARG2 proteins were similar between asthmatics on corticosteroids and those not on corticosteroids (all *P* > 0.05)(S2 Table). As previously reported [4, 5], F_E_NO was inversely related to lung function in asthma [F_E_NO correlation to, FEV_1_% predicted, *R* = -0.414, *P* = 0.003; FEV_1_/FVC, *R* = -0.311, *P* = 0.02; PC_20_, *R* = -0.469, *P* = 0.01](Table 2). IgE levels in asthmatics were positively correlated with the expression of ARG2 and iNOS in the airway epithelium [IgE correlation to, ARG2, *R* = 0.730, *P* = 0.02; iNOS, *R* = 0.624, *P* = 0.05](Table 2). ARG2 expression and arginase activity were not related to lung function in asthmatics (all *P* > 0.05)(Table 2), which is consistent with our prior studies [5, 6]. F_E_NO levels were inversely related to lung function in individuals grouped by mild or severe asthma (*P* < 0.05), but not in individuals with moderate asthma (*P* > 0.05)(S3 Table). In asthmatics on corticosteroids, F_E_NO levels were inversely related to lung function (*P* < 0.05). IgE levels were positively correlated with the expression of iNOS in the airway epithelium in asthmatics not on corticosteroids (*P* = 0.01)(S4 Table).

**Table 2:** Correlation between arginine metabolic endotype and clinical asthma sub-phenotype.

Characteristics	Statistics*	FEV1 % predicted	FEV_1_/FVC	IgE IU/ml	PC20 mg/ml
F_E_NO, ppb	*R*	**-0.414**	**-0.311**	0.242	**-0.469**
	*P*	**0.003**	**0.02**	0.18	**0.01**
iNOS/CK	*R*	0.184	0.327	**0.624**	-0.082
	*P*	0.6	0.3	**0.05**	0.8
Arginase activity, μmol/ml/h	*R*	-0.011	-0.093	0.418	-0.083
	*P*	0.9	0.7	0.15	0.8
ARG2/CK	*R*	0.400	0.226	**0.730**	0.089
	*P*	0.2	0.5	**0.02**	0.8

Definition of abbreviations: FEV_1_, Forced expiratory volume in 1 second; FVC, Forced vital capacity; PC_20_, provocative concentration of methacholine causing a 20% fall in FEV_1_; F_E_NO, fractional exhaled nitric oxide; iNOS, inducible nitric oxide synthase; CK, Cytokeratin; ARG2, arginase 2; iNOS/CK and ARG2/CK determined in the airway epithelium;

*R and *P* values represent Multivariate Pairwise correlation and significance, respectively; Values in bold indicate *R* values with significant *P* ≤ 0.05.

**Fig 1:**
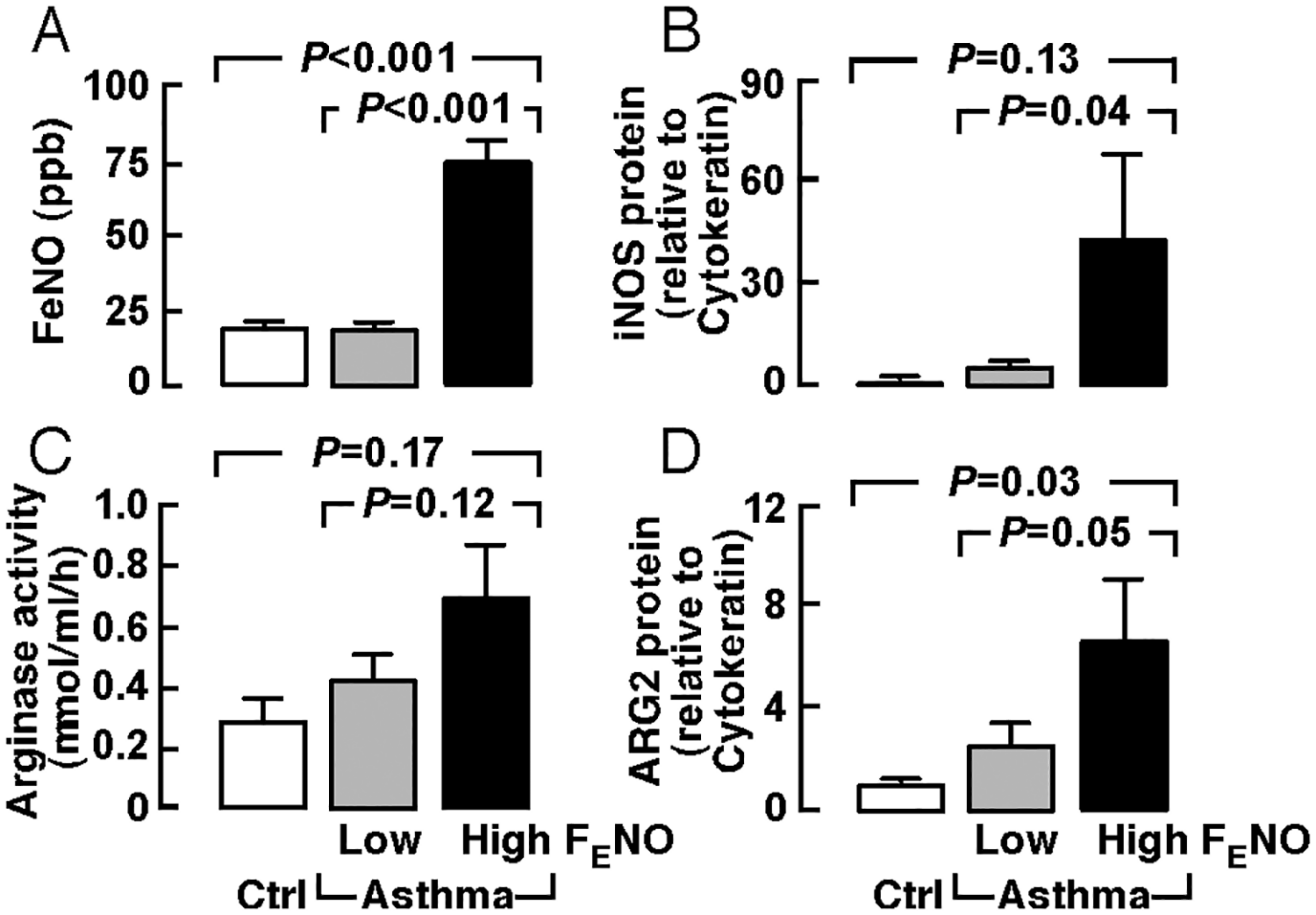
Arginine metabolic endotype of asthma. Fractional exhaled nitric oxide (F_E_NO), serum arginase activity, and airway protein expression of inducible nitric oxide synthase (iNOS) and arginase 2 (ARG2) were dichotomized by high F_E_NO (≥ 35 ppb, n = 23) and low F_E_NO (< 35 ppb, *n* = 29).

Asthmatics had higher levels of F_E_NO than controls (*P* < 0.001), but 56% of asthmatics actually had F_E_NO within the normal range. When we stratified asthmatics by high and low F_E_NO [high F_E_NO, ≥ 35 ppb, *n* = 23, low F_E_NO, < 35 ppb, *n* = 29] [4, 14], asthmatics with high F_E_NO had higher expression of iNOS (*P* = 0.04) and ARG2 (*P* = 0.05) in the airway epithelium (Table 3)(Fig 1). These data suggest that F_E_NO can be used as a marker of high arginine metabolism in asthmatic airways. Serum arginase activity, although higher in asthmatics, was not significantly different in asthmatics with high or low F_E_NO (*P* = 0.12)(Table 3)(Fig 1). With all three severe asthmatics stratified to high F_E_NO phenotype, high F_E_NO asthmatics tended to be more severe than low F_E_NO asthmatics (Asthma severity, mild/moderate/severe, low F_E_NO, 17/11/0, high F_E_NO, 10/7/3, *P* = 0.06)(Table 3). Because arginine metabolism influences cellular bioenergetics [6, 20], we measured the concentration of lactate in the bronchoalveolar lavage (BAL). Asthmatics with high F_E_NO had higher levels of lactate in the BAL (ANOVA *P* = 0.02), lower FEV_1_% predicted (ANOVA *P* < 0.001), lower FEV_1_/FVC (ANOVA *P* = 0.0002) and lower PC_20_ (ANOVA *P* < 0.001) as compared to low F_E_NO asthmatics or healthy controls. Importantly, asthmatics with low FeNO, whose airway expression of iNOS and ARG2 were similar to healthy controls (all *P* > 0.05), had significantly higher PC_20_ (*P* < 0.001) and lower IgE (*P* = 0.04) as compared to high F_E_NO asthmatics (Table 3)(Fig 1). There was no difference in proportion of asthmatics receiving corticosteroids or not on corticosteroids between high F_E_NO and low F_E_NO groups (*P* > 0.05)(Table 3).

**Table 3:** Features in healthy controls and asthmatics stratified by NO levels.

Characteristics	Healthy Controls	Asthmatics			P**
		Low F_E_NO (< 35 ppb)	High F_E_NO (≥ 35 ppb)	P*	
N	51	29	23		
Mean age, yr	37 ± 1	38 ± 1	38 ± 2	0.9	0.9
Gender, M/F	18/33	12/17	11/12	0.6	0.5
BMI	27.8 ± 0.9	28.5 ± 1.1	29.5 ± 1.0	0.5	0.5
Ethnicity, C/AA/other	24/23/4	15/11/3	10/9/4	0.8	0.7
Asthma Severity					
Mild/Moderate/Severe		17/11/0	10/7/3	0.06	
Lung function					
FEV_1_% predicted	96 ± 1	86 ± 3	78 ± 3	0.04	<0.001
FEV_1_/FVC	0.80 ± 0.01	0.76 ± 0.01	0.72 ± 0.02	0.11	0.0002
PC_20_, mg/ml	NR^#^	8.2 ± 2.2	0.9 ± 0.2	<0.001	<0.001
F_E_NO, ppb	19 ± 1	19 ± 1	75 ± 7	<0.001	<0.001
Blood					
IgE, IU/ml	76 ± 21	221 ± 60	377 ± 84	0.04	0.0002
Arginase activity, μmol/ml/h	0.30 ± 0.07	0.43 ± 0.08	0.70 ± 0.17	0.12	0.17
BAL					
Lactate, μM	11 ± 1	11 ± 1	25 ± 6	0.01	0.02
Airway epithelium					
iNOS/CK	1.1 ± 0.2	4.9 ± 2.0	42.8 ± 25.9	0.04	0.13
ARG2/CK	1.0 ± 0.1	2.5 ± 1.0	6.6 ± 2.3	0.05	0.03
Medication (*n*)					
Corticosteroids (no/yes)	51/0	11/18	11/12	0.4	
Inhaled (no/yes)	51/0	11/18	11/12	0.4	
Oral (no/yes)	51/0	29/0	23/0		
Injected (no/yes)	51/0	29/0	23/0		
Xolair (no/yes)	51/0	29/0	23/0		
Leukotriene receptor antagonists (no/yes)	51/0	23/6	18/5	0.9	

Mean ± SEM;

Definition of abbreviations: M, male; F, female; BMI, body mass index; C, Caucasian; AA, African Amirican; Mild, mild intermittent/persistent; Moderate, moderate persistent; Severe, severe persistent; FEV_1_, Forced expiratory volume in 1 second; FVC, Forced vital capacity; PC_20_, provocative concentration of methacholine causing a 20% fall in FEV_1_; F_E_NO, fractional exhaled nitric oxide; BAL, bronchoalveolar lavage; iNOS, inducible nitric oxide synthase; CK, Cytokeratin; ARG2, arginase 2;

*P value, asthmatics with high F_E_NO *vs*. asthmatics with low F_E_NO;

**P value, ANOVA of asthmatics with high F_E_NO, asthmatics with low F_E_NO, controls;

^#^non-reactive.

These findings indicate that high arginine metabolism in asthma is associated with the high F_E_NO phenotype, which is characterized clinically by greater airflow obstruction and airway reactivity and more severe asthma.

### Greater reliance on glycolysis in bronchial epithelial cells with iNOS overexpression

**Fig 2:**
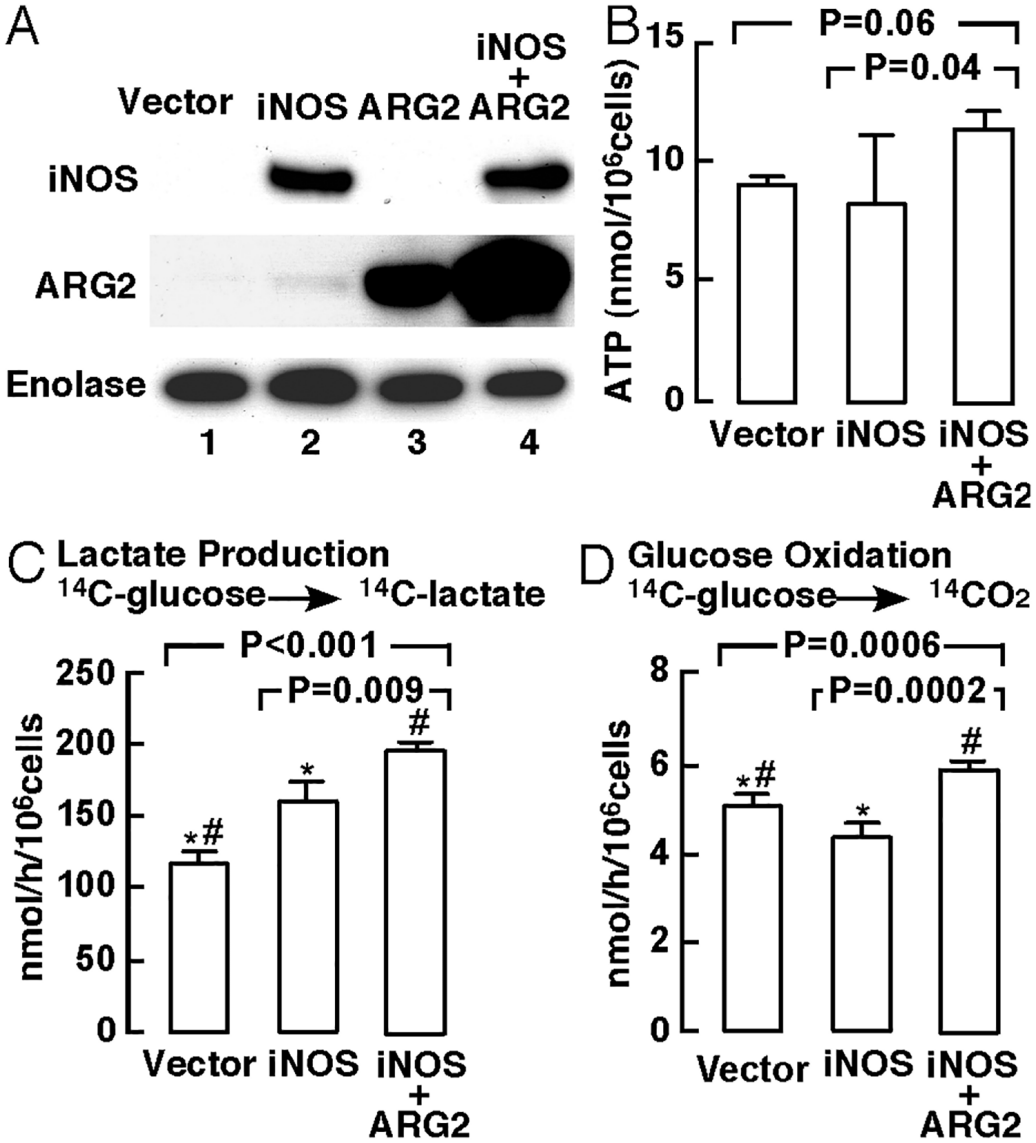
Protein expression and bioenergetics of bronchial epithelial cells with iNOS expression. (A) Western analyses of BET1A cells transfected with iNOS vector, ARG2 vector, co-transfected with iNOS+ARG2, or control vector (n ≥ 3 replicate experiments). Enolase was used as a loading control. (B) ATP production determined in BET1A cells transfected with iNOS vector, co-transfected with iNOS+ARG2, or control vector (n ≥ 3 replicate experiments). (C–D) Radioisotope studies of glucose metabolism in BET1A cells with iNOS expression. The rates of production of lactate (C) and oxidation of glucose (D) using radioactive glucose tracer were analyzed in BET1A cells transfected with iNOS vector, control vector, or co-transfected with iNOS+ARG2 vectors (n ≥ 3 replicate experiments). *P < 0.05, iNOS-expressing cells *vs*. control vector-transfected cells; #*P* < 0.05, iNOS+ARG2 co-transfected cells *vs*. control-transfected cells.

In our recent study [6], we reported that greater arginine flux through ARG2 can support cellular mitochondrial bioenergetics. In order to understand the impact of increased arginine metabolism *via* high expression of iNOS or high co-expression of iNOS and ARG2 in asthmatic bronchial epithelial cells, we analyzed immortalized human bronchial epithelial cells (BET1A) *in vitro*. BET1A cells were transiently transfected with iNOS expression vector, ARG2 expression vector, co-transfected with iNOS vector and ARG2 vector (iNOS+ARG2), or control vector. iNOS expression is not detectable in BET1A cells at baseline culture [21]. When transfected with iNOS vector alone or co-transfected with iNOS and ARG2 vectors, high-level expression of iNOS and ARG2 were observed (Fig 2A). High nitrate levels accumulated in the culture medium of cells transfected with iNOS vector or with iNOS+ARG2 vectors, and this was suppressed by N-nitroarginine methyl ester (L-NAME), a non-selective inhibitor of NO synthase, confirming iNOS activity [nitrate μM, vector alone 2.6 ± 0.1, *n* = 26, iNOS 52.5 ± 3.3, *n* = 15, iNOS+L-NAME 3.6 ± 0.2, *n* = 4, iNOS+ARG2 41.0 ± 3.3, *n* = 22, iNOS+ARG2+L-NAME 5.6 ± 0.9, *n* = 9, ANOVA *P* < 0.001].

The effect of iNOS on bioenergetics of BET1A cells was assessed by quantifying the rate of production of ATP and metabolism of glucose using radioactive tracer (*n* ≥ 3 replicate experiments)(Fig 2B–2D). BET1A cells transfected with iNOS+ARG2 had a trend towards higher production of ATP [nmol/10^6^ cells, vector alone 9.1 ± 0.3, *n* = 8, iNOS 8.2 ± 2.2, *n* = 4, iNOS+ARG2 11.4 ± 0.7, *n* = 10, ANOVA *P* = 0.06](Fig 2B). The production of ATP was significantly higher by cells transfected with iNOS+ARG2 when compared with those transfected with iNOS alone (*P* = 0.04). Both iNOS transfected cells (*P* < 0.001) and iNOS+ARG2 transfected cells (*P* = 0.0002) had high rate of glycolysis, as evidenced by high rate of production of [^14^C]lactate from [^14^C]glucose, as compared with vector alone [lactate production nmol/h/10^6^ cells, vector alone 117 ± 8, *n* = 18, iNOS 159 ± 14, *n* = 9, iNOS+ARG2 195 ± 4 *n* = 16, ANOVA *P* < 0.001]. The rate of oxidation of glucose was lower in the iNOS alone expressing cells (*P* = 0.04) and was higher in the iNOS+ARG2 co-transfected cells (*P* = 0.01)[glucose oxidation nmol/h/10^6^ cells, vector alone 5.0 ± 0.2, *n* = 18, iNOS 4.3 ± 0.2, *n* = 9, iNOS+ARG2 5.8 ± 0.1, *n* = 16, ANOVA *P* = 0.0006](Fig 2C and 2D). Parallel experiments were performed for the measurement of oxygen consumption rate (OCR) using Seahorse XF system (*n* ≥ 3 replicate experiments). As compared with vector alone, iNOS transfected cells had a trend towards lower basal OCR (basal OCR pmol O_2_/min, vector alone 132 ± 16, *n* = 13, iNOS 103 ± 8, *n* = 13, *P* = 0.09), and significantly lower coupling efficiency, which is defined as the proportion of the oxygen consumed to drive ATP synthesis compared to proton leak (coupling efficiency, %, vector alone 82 ± 3, *n* = 13, iNOS 75 ± 2, *n* = 13, *P* = 0.04)(S1 Fig) [22]. Cells co-transfected with ARG2+iNOS had similar basal OCR (*P* > 0.05) and coupling efficiency (*P* > 0.05) as compared to vector alone. Both iNOS transfected cells (*P* = 0.02) and iNOS+ARG2 co-transfected cells (*P* = 0.04) had lower spare respiratory capacity than control vector-transfected cells (S1 Fig). Taken together, these data suggest a higher rate of aerobic glycolysis by iNOS transfected cells that was ameliorated by co-expression of ARG2.

## Discussion

The presence of high levels of iNOS, which produces NO and citrulline from arginine, and of arginase, which converts arginine to ornithine and urea, suggests that some asthma phenotypes are in a state of very high arginine catabolism. Important to identification of this high arginine metabolic sub-phenotype, we found that asthmatics with high F_E_NO had higher expression of iNOS and higher expression of ARG2 in the airway epithelium than the low F_E_NO group. As compared to low F_E_NO asthmatics, high F_E_NO asthmatics had a more severe clinical phenotype, which confirms prior report of high F_E_NO being an at-risk phenotype for reactivity and exacerbations of asthma [4]. The high F_E_NO group had higher airway lactate levels, suggesting other metabolic changes may be present in this group. The *in vitro* studies of immortalized bronchial epithelial cells showed that high co-expression of iNOS and ARG2 shifted cellular metabolism to greater oxidative metabolism of glucose and higher rate of ATP production. Altogether, the high F_E_NO group is characterized by greater arginine metabolism and greater oxidative glucose metabolism, which is associated with a more severe clinical phenotype.

Inhaled corticosteroids are considered to be the most effective medications for asthma control. Previously we reported suppressive effects of corticosteroids on iNOS induction [1]. We [14, 23] and others [24–27] have shown that F_E_NO levels decrease when asthmatics are treated with corticosteroids. Here, asthmatics receiving inhaled corticosteroids tended to have lower F_E_NO levels than asthmatics not on corticosteroids, however, there was no difference in proportion of asthmatics receiving corticosteroids or not on corticosteroids between high F_E_NO and low F_E_NO groups, suggesting that high F_E_NO and low F_E_NO groups are phenotypes which represent arginine metabolic endotypes and not only a reflection of corticosteroid use.

The greater reliance of iNOS-expressing cells on glycolysis has been reported previously. NO binds to several targets and inhibits their functions within the mitochondrial respiratory chain (e.g., complexes I, III and IV)[28–31]. Inhibition of the respiratory chain by NO consequently decreases oxygen consumption and cellular respiration and results in an increase in the rate of aerobic glycolysis and an increase in the production of pyruvate and lactate [31, 32]. Here, iNOS transfected cells had greater lactate production, significantly reduced coupling efficiency and spare respiratory capacity, most likely as a result of the inhibition of cellular respiration by NO. Expression of ARG2 reversed suppressive effects of iNOS/NO on glucose oxidative metabolism, which suggests that ARG2 arginine metabolism may protect against some of the adverse effects of NO on mitochondrial respiratory function.

Many studies support a link between metabolism and asthma. Prior studies have identified metabolic changes in the airway in the murine model of asthma [33]. Furthermore, mitochondria numbers and oxygen consumption in airway smooth muscle of asthmatics are greater than in healthy controls [34]. Platelets from asthmatic individuals have less reliance on glycolysis and greater tricarboxylic acid (TCA) cycle turnover [35]. Greater arginine flux through ARG2 in the mitochondria has recently been shown to drive TCA cycle and cellular respiration [6]. Metabolism of arginine regulates T cell function and fate, with higher levels of arginine increasing T cell survival and anti-tumor responses [20]. Thus, this work suggests that identification of the high NO sub-phenotype of asthma may enable strategies to target metabolic pathways for personalized asthma care.

Arginine bioavailability may impact nitric oxide production and potentially F_E_NO. We previously reported an increase in whole body metabolism of arginine in asthmatics as compared to healthy controls using stable isotope–labeled tracers [6]. Extracellular arginine levels do not reflect arginine metabolism or its cellular compartmentalization. Arginine is taken into cells by cationic amino acid transporter (CAT) proteins, and arginine and other urea cycle amino acids are highly compartmentalized in tissues and within intracellular pools, and do not equilibrate rapidly with extracellular pools. A recent study showed that intracellular arginine is critical in the control of glycolysis and mitochondrial activity [20], and intracellular arginine flux and mitochondrial arginine metabolism is a critical determinant of cell bioenergetics and function [6, 20]. In the present study, we assessed airway epithelial enzyme levels and airway exhaled NO. However, we did not evaluate plasma arginine and arginine/ornithine ratios, or intracellular arginine.

Taken together, this study shows that the high F_E_NO phenotype represents a greater arginine metabolism endotype that is clinically characterized by greater airflow obstruction and airway reactivity. This new understanding is important to plan metabolic interventions and provide therapeutic options separate from corticosteroids and other anti-inflammatories.

## Supporting information

S1 Table Aginine metabolic endotype of asthmatics based on asthma severity. (DOCX)

S2 Table Aginine metabolic endotype of asthmatics based on corticosteroids use. (DOCX)

S3 Table Correlation between arginine metabolic endotype and clinical asthma sub-phenotype based on asthma severity. (DOCX)

S4 Table Correlation between arginine metabolic endotype and clinical asthma sub-phenotype based on corticosteroids use. (DOCX)

S1 Fig The oxygen consumption rate (OCR) of BET1A cells with iNOS expression. Basal OCR (A), coupling efficiency (B) and spare respiratory capacity (C) were analyzed in BET1A cells transfected with iNOS vector, co-transfected with iNOS+ARG2, or control vector (n ≥ 3 replicate experiments). *P < 0.05, iNOS-expressing cells *vs*. control vector-transfected cells; #*P* < 0.05, iNOS+ARG2 co-transfected cells *vs*. control-transfected cells.
(TIF)
