# Supplementary material for: Arginine metabolic endotypes related to asthma severity
Source: PLoS One. 2017 Aug 10;12(8):e0183066. doi: 10.1371/journal.pone.0183066 (PMC5552347; doi:10.1371/journal.pone.0183066)
Supplement: S1 Table — (DOCX) [file pone.0183066.s001.docx]

**S1 Table. Aginine metabolic endotype of asthmatics based on asthma severity**

| **Characteristics** | **Healthy Controls** | **All**  **Asthmatics** | ***P**** | **Asthma Severity** | | | | |
| --- | --- | --- | --- | --- | --- | --- | --- | --- |
|  |  |  |  | **Mild** | **Moderate** | **Severe** | ***P***** |  |
| F_E_NO, ppb | 19 ± 1 | 43 ± 5 | <0.001 | 33 ± 5 | 46 ± 9 | 108 ± 36 | 0.003 |  |
| iNOS/CK | 1.1 ± 0.2 | 23.9 ± 13.8 | 0.01 | 37.4 ± 27.0 | 4.8 ± 2.6 | 32.5 | 0.5 |  |
| Arginase activity, μmol/ml/h | 0.30 ± 0.07 | 0.52 ± 0.08 | 0.05 | 0.55 ± 0.09 | 0.49 ± 0.15 |  | 0.7 |  |
| ARG2/CK | 1.0 ± 0.1 | 3.0 ± 1.3 | 0.03 | 6.2 ± 2.4 | 2.2 ± 1.2 | 5.85 | 0.3 |  |

Mean ± SEM;

Definition of abbreviations: Mild, mild intermittent/persistent; Moderate, moderate persistent; Severe, severe persistent; F_E_NO, fractional exhaled nitric oxide; iNOS, inducible nitric oxide synthase; CK, Cytokeratin; ARG2, arginase 2; iNOS/CK and ARG2/CK determined in the airway epithelium;

**P* value, asthma *vs.* controls; ***P* value, ANOVA of mild, moderate, severe asthma.
