# Supplementary material for: Arginine metabolic endotypes related to asthma severity
Source: PLoS One. 2017 Aug 10;12(8):e0183066. doi: 10.1371/journal.pone.0183066 (PMC5552347; doi:10.1371/journal.pone.0183066)
Supplement: S2 Table — (DOCX) [file pone.0183066.s002.docx]

**S2 Table. Aginine metabolic endotype of asthmatics based on corticosteroids use**

| **Characteristics** | **Corticosteroids** | | ***P**** |
| --- | --- | --- | --- |
|  | **no** | **yes** |  |
| F_E_NO, ppb | 45 ± 6 | 42 ± 7 | 0.17 |
| iNOS/CK | 53.1 ± 45.9 | 11.3 ± 4.5 | 0.4 |
| Arginase activity, μmol/ml/h | 0.62 ± 0.11 | 0.47 ± 0.12 | 0.3 |
| ARG2/CK | 4.5 ± 3.8 | 4.3 ± 1.4 | 0.9 |

Mean ± SEM;

Definition of abbreviations: F_E_NO, fractional exhaled nitric oxide; iNOS, inducible nitric oxide synthase; CK, Cytokeratin; ARG2, arginase 2; iNOS/CK and ARG2/CK determined in the airway epithelium;

**P* value, asthmatics not on corticosteroids *vs.* on corticosteroids.
