# Supplementary material for: Arginine metabolic endotypes related to asthma severity
Source: PLoS One. 2017 Aug 10;12(8):e0183066. doi: 10.1371/journal.pone.0183066 (PMC5552347; doi:10.1371/journal.pone.0183066)
Supplement: S3 Table — (DOCX) [file pone.0183066.s003.docx]

**S3 Table. Correlation between arginine metabolic endotype and clinical asthma sub-phenotype based on asthma severity**

| **Characteristics** | **Asthma**  **Sevetity** | **Statistics*** | **FEV1**  **% predicted** | **FEV_1_/FVC** | **IgE**  **IU/ml** | **PC20**  **mg/ml** |
| --- | --- | --- | --- | --- | --- | --- |
| F_E_NO, ppb | Mild | *R* | **-0.408** | 0.009 | 0.272 | **-0.515** |
|  |  | *P* | **0.03** | 0.9 | 0.2 | **0.04** |
|  | Moderate | *R* | 0.357 | 0.010 | 0.322 | -0.426 |
|  |  | *P* | 0.14 | 0.9 | 0.3 | 0.19 |
|  | Severe | *R* | -0.934 | **-0.998** |  |  |
|  |  | *P* | 0.2 | **0.04** |  |  |
| iNOS/CK | Mild | *R* | -0.361 | 0.469 | 0.643 | -0.182 |
|  |  | *P* | 0.5 | 0.4 | 0.2 | 0.7 |
|  | Moderate | *R* | -0.074 | -0.006 | -0.517 |  |
|  |  | *P* | 0.9 | 0.9 | 0.4 |  |
|  | Severe | *R* |  |  |  |  |
|  |  | *P* |  |  |  |  |
| Arginase activity, μmol/ml/h | Mild | *R* | -0.664 | -0.407 | 0.602 | -0.302 |
|  |  | *P* | 0.10 | 0.3 | 0.2 | 0.5 |
|  | Moderate | *R* | 0.243 | -0.104 | 0.311 | -0.156 |
|  |  | *P* | 0.5 | 0.8 | 0.4 | 0.8 |
|  | Severe | *R* |  |  |  |  |
|  |  | *P* |  |  |  |  |
| ARG2/CK | Mild | *R* | -0.160 | -0.369 | 0.919 | -0.143 |
|  |  | *P* | 0.8 | 0.6 | 0.08 | 0.8 |
|  | Moderate | *R* | 0.878 | 0.829 | -0.422 |  |
|  |  | *P* | 0.12 | 0.17 | 0.5 |  |
|  | Severe | *R* |  |  |  |  |
|  |  | *P* |  |  |  |  |

Definition of abbreviations: FEV_1_, Forced expiratory volume in 1 second; FVC, Forced vital capacity; PC_20_, provocative concentration of methacholine causing a 20% fall in FEV_1_; F_E_NO, fractional exhaled nitric oxide; iNOS, inducible nitric oxide synthase; CK, Cytokeratin; ARG2, arginase 2; iNOS/CK and ARG2/CK determined in the airway epithelium; Mild, mild intermittent/persistent; Moderate, moderate persistent; Severe, severe persistent;

**R* and *P* values represent Multivariate Pairwise correlation and significance, respectively; Values in bold indicate *R* values with significant *P* ≤ 0.05.
