# Supplementary material for: Arginine metabolic endotypes related to asthma severity
Source: PLoS One. 2017 Aug 10;12(8):e0183066. doi: 10.1371/journal.pone.0183066 (PMC5552347; doi:10.1371/journal.pone.0183066)
Supplement: S4 Table — (DOCX) [file pone.0183066.s004.docx]

**S4 Table. Correlation between arginine metabolic endotype and clinical asthma sub-phenotype based on corticosteroids use**

| **Characteristics** | **Corticosteroids** | **Statistics*** | **FEV1**  **% predicted** | **FEV_1_/FVC** | **IgE**  **IU/ml** | **PC20**  **mg/ml** |
| --- | --- | --- | --- | --- | --- | --- |
| F_E_NO, ppb | no | *R* | -0.337 | -0.280 | 0.200 | -0.489 |
|  |  | *P* | 0.17 | 0.2 | 0.5 | 0.12 |
|  | yes | *R* | **-0.463** | **-0.367** | 0.224 | **-0.487** |
|  |  | *P* | **0.009** | **0.04** | 0.3 | **0.04** |
| iNOS/CK | no | *R* | -0.555 | 0.448 | **0.999** | 0.970 |
|  |  | *P* | 0.6 | 0.7 | **0.01** | 0.15 |
|  | yes | *R* | -0.086 | -0.381 | 0.444 | -0.466 |
|  |  | *P* | 0.8 | 0.3 | 0.3 | 0.4 |
| Arginase activity, μmol/ml/h | no | *R* | 0.179 | 0.323 | -0.071 | **0.957** |
|  |  | *P* | 0.7 | 0.5 | 0.9 | **0.04** |
|  | yes | *R* | -0.123 | -0.425 | 0.569 | -0.049 |
|  |  | *P* | 0.7 | 0. | 0.10 | 0.9 |
| ARG2/CK | no | *R* |  |  |  |  |
|  |  | *P* |  |  |  |  |
|  | yes | *R* | 0.489 | 0.372 | 0.658 | 0.038 |
|  |  | *P* | 0.2 | 0.4 | 0.10 | 0.9 |

Definition of abbreviations: FEV_1_, Forced expiratory volume in 1 second; FVC, Forced vital capacity; PC_20_, provocative concentration of methacholine causing a 20% fall in FEV_1_; F_E_NO, fractional exhaled nitric oxide; iNOS, inducible nitric oxide synthase; CK, Cytokeratin; ARG2, arginase 2; iNOS/CK and ARG2/CK determined in the airway epithelium;

**R* and *P* values represent Multivariate Pairwise correlation and significance, respectively; Values in bold indicate *R* values with significant *P* ≤ 0.05.
