# Supplementary figures and images for: Arginine metabolic endotypes related to asthma severity
Source: PLoS One. 2017 Aug 10;12(8):e0183066. doi: 10.1371/journal.pone.0183066 (PMC5552347; doi:10.1371/journal.pone.0183066)

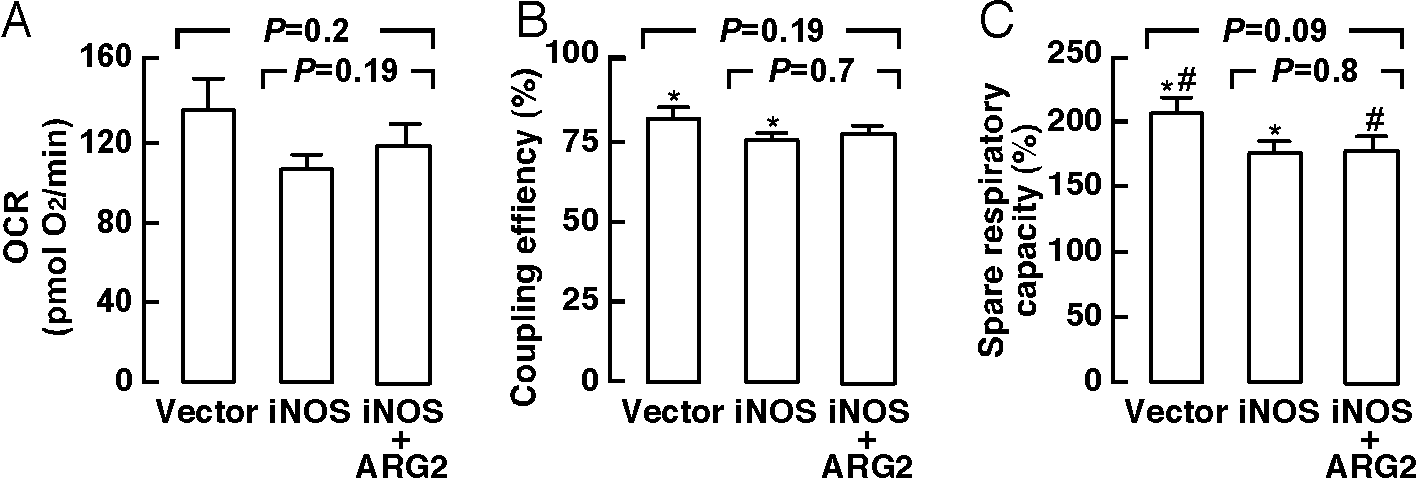

Supplement: S1 Fig — Basal OCR (A), coupling efficiency (B) and spare respiratory capacity (C) were analyzed in BET1A cells transfected with iNOS vector, co-transfected with iNOS+ARG2, or control vector (n ≥ 3 replicate experiments). *P < 0.05, iNOS-expressing cells vs. control vector-transfected cells; #P < 0.05, iNOS+ARG2 co-transfected cells vs. control-transfected cells. (TIF) [file pone.0183066.s005.tif]
